# Synchrony in Parkinson's disease: importance of intrinsic properties of the external globus pallidus

**DOI:** 10.3389/fnsys.2013.00060

**Published:** 2013-10-04

**Authors:** Bettina C. Schwab, Tjitske Heida, Yan Zhao, Enrico Marani, Stephan A. van Gils, Richard J. A. van Wezel

**Affiliations:** ^1^Applied Analysis and Mathematical Physics, MIRA Institute of Technical Medicine and Biomedical Technology, University of TwenteEnschede, Netherlands; ^2^Biomedical Signals and Systems, MIRA Institute of Technical Medicine and Biomedical Technology, University of TwenteEnschede, Netherlands; ^3^Biophysics, Donders Institute for Brain, Cognition and Behavior, Radboud UniversityNijmegen, Netherlands

**Keywords:** external globus pallidus, dopamine depletion, desynchronization, plasticity, GPe-GPe synapses, HCN channels, SK channels, NaF channels

## Abstract

The mechanisms for the emergence and transmission of synchronized oscillations in Parkinson's disease, which are potentially causal to motor deficits, remain debated. Aside from the motor cortex and the subthalamic nucleus, the external globus pallidus (GPe) has been shown to be essential for the maintenance of these oscillations and plays a major role in sculpting neural network activity in the basal ganglia (BG). While neural activity of the healthy GPe shows almost no correlations between pairs of neurons, prominent synchronization in the β frequency band arises after dopamine depletion. Several studies have proposed that this shift is due to network interactions between the different BG nuclei, including the GPe. However, recent studies demonstrate an important role for the properties of neurons within the GPe. In this review, we will discuss these intrinsic GPe properties and review proposed mechanisms for activity decorrelation within the dopamine-intact GPe. Failure of the GPe to desynchronize correlated inputs can be a possible explanation for synchronization in the whole BG. Potential triggers of synchronization involve the enhancement of GPe-GPe inhibition and changes in ion channel function in GPe neurons.

## 1. Introduction

Neural activity in the basal ganglia (BG) of patients with idiopathic Parkinson's disease (PD) and animal models of PD commonly shows high levels of synchronization, bursting, and oscillations in low frequency bands such as θ (4–7 Hz) and β (15–30 Hz) frequencies (Bergman et al., 1994; Obeso et al., 2000; Brown et al., 2001; Montgomery, 2007; Wichmann et al., 2011). Although it is not completely clear whether these abnormal neural activities cause PD motor symptoms, they are reliable disease markers as they coincide with motor symptoms after severe dopamine depletion (Kühn et al., 2006, 2009; Hammond et al., 2007; Eusebio et al., 2007; Quiroga-Varela et al., 2013). Nevertheless, the mechanisms and origins of the emergence and transmission of synchronization, bursting and oscillations remain controversial. Oscillations in the β frequency range, often related to rigidity, akinesia and bradykinesia, have been proposed to arise via the cortex (Brown, 2003; Sharott et al., 2005; Tachibana et al., 2011) or via interactions of the subthalamic nucleus (STN) and the external globus pallidus (GPe) (Plenz and Kital, 1999; Bevan et al., 2002; Terman et al., 2002; Tachibana et al., 2011; Fan et al., 2013).

After dopamine depletion, prominent changes in neural synchronization occur in projection neurons of the GPe, which has a central position in the BG loop (Smith et al., 1998)^1^. Under heathy conditions, activity in the GPe shows almost no correlations between pairs of neurons (Nini et al., 1995; Raz et al., 2000; Mallet et al., 2008), including spatially nearby neurons (Bar-Gad et al., 2003), although neurons in the GPe possess a large number of local axon collaterals and are believed to receive common inputs (Francois et al., 1984; Percheron et al., 1991; Yelnik, 2002). In contrast, after dopamine depletion, strong synchronization in the β frequency range was found (Nini et al., 1995; Raz et al., 2000; Heimer et al., 2002; Mallet et al., 2008). These findings led to the suggestion of a local mechanism that decorrelates activity in the healthy GPe (Bar-Gad et al., 2003). Failure of the GPe to decorrelate synchronized input can be an explanation for abnormal synchrony of the whole BG.

In this review, we discuss recent evidence supporting the crucial role of GPe properties in synchronizing and desynchronizing afferent activity and their remodeling during parkinsonism. We describe proposed mechanisms for this synchronization process intrinsic to the GPe, based on synaptic and cellular properties.

## 2. Intrinsic GPe structure

The GPe is located centrally in the BG and essentially contributes to its multiple feedback loops (Jaeger and Kita, 2011). Its neural dynamics, involving high firing rates, are strongly influenced by excitatory inputs from the STN (Goldberg et al., 2003). GABAergic synapses projecting to the GPe are provided mainly by the striatum and about 10% of the synapses arise in the GPe itself (Shink and Smith, 1995). Morphological characterization of these local axon collaterals in the rat brain indicates that the GP not only acts as a relay nucleus, but has intrinsic structures capable of internal information processing (Sadek et al., 2007). In these structures, information is processed from neurons in the outer part of the GP to neurons in the inner part (Sadek et al., 2007). This elaborate GP internal connectivity seems essential for sculpting GP activity, and GP projection neurons may take additional roles as inhibitory interneurons that control spiking behavior.

In healthy awake animals, two electrophysiological cell types have been identified in the GPe based on their firing rates and patterns (deLong, 1971; Bugaysen et al., 2010; Benhamou et al., 2012). 6-hydroxydopamine (6-OHDA) treated rats also showed clear differences in the firing rates and patterns between two distinct GP neuron populations *in vivo* (Mallet et al., 2008). In contrast, studies using healthy rat brain slices described three electrophysiological subgroups of neurons in the GP (Cooper and Stanford, 2000; Bugaysen et al., 2010). However, more recent *in vitro* studies reported no clear qualitative electrophysiological differences amongst GP neurons and challenge the existence of distinct GP neuron types (Chan et al., 2004, 2011; Hashimoto and Kita, 2006; Günay et al., 2008; Deister et al., 2013).

Nevertheless, anatomical dichotomy has often been described in the GP (Cooper and Stanford, 2002; Hoover and Marshall, 2002; Nobrega-Pereira et al., 2010). For instance, a group of proenkephalin positive neurons that preferentially target the striatum (Hoover and Marshall, 2002) and a small population of calretinin positive interneurons (Cooper and Stanford, 2002) have been reported. Based on fate mapping analysis, even five neural populations have been identified in the mouse GP which differ in progenitor lineage and partly in their embryonic domains (Nobrega-Pereira et al., 2010).

Recently, Mallet et al. (2012) combined anatomical and electrophysiological characteristics of classes of GP neurons. They described the existence of two distinct neural populations in the GP of a 6-OHDA treated rat that have distinct molecular profiles and axonal connectivities. Neurons of the first population fired antiphasic to STN neurons, often expressed parvalbumin (PV) and targeted the STN or the EP. The second population described a novel cell type: neurons that fired in-phase with STN neurons expressed proenkephalin and innervated both projection neurons and interneurons of the striatum. Mallet et al. (2012) also found differences in the dendritic and axonal architectures of the two cell types. In particular, local axon collaterals of the first neural population were longer while their dendritic spine density was lower in comparison to the second population.

Altogether, the complex structure of the GPe on both the synaptic and cellular levels indicates that information processing within the GPe is possible and might be critical for modulating dynamics in the whole BG network.

## 3. Important contribution of the GPe to the pathophysiology of parkinsonism

The GPe is in a unique position to propagate synchronized oscillatory activity, since it projects to virtually all other BG nuclei (Mallet et al., 2008). Furthermore, its neurons possess intrinsic oscillatory properties, leading to a steady pacemaking function (Wilson, 2013). Nevertheless, β band oscillations in the GPe in parkinsonism commonly exhibit smaller amplitudes than those in the STN or the GPi (Stein and Bar-Gad, 2013).

An important hypothesis proposes that the GPe plays a major role in information processing in the dopamine depleted BG, in particular by interacting with the STN (Plenz and Kital, 1999; Bevan et al., 2002; Terman et al., 2002; Fan et al., 2013). A study in 1-Methyl-4-Phenyl-1,2,3,6-Tetrahydropyridine (MPTP) treated monkeys showed that muscimol inactivation of the GPe to block its GABAergic outputs led to prominent reductions of β oscillations in the STN (Tachibana et al., 2011). The GPe is therefore assumed to regulate the presence of oscillations in the dopamine depleted BG, while the origins of these oscillations remain unclear.

Since neural activity abnormalities in PD are reversible with L-3,4-dihydroxyphenylalanine (L-Dopa) treatment, their emergence and reversal are both thought to be crucially dependent on dopamine levels, although possibly involving different mechanisms (Brown et al., 2001; Kühn et al., 2006; Tachibana et al., 2011). Despite that in literature the effects of dopamine depletion are often focused on the striatum, PD patients lose about 82% of dopamine in the GPe (Rajput et al., 2008). This specific loss is also attributed to motor symptoms as supported by several studies investigating the influence of dopamine directly in the rat GP. Firstly, dopamine receptor *D*_1_/*D*_2_ blockage in the GP induced akinesia (Hauber and Lutz, 1999). Secondly, direct application of dopamine in the GP restored motor behavior in a 6-OHDA model (Galvan et al., 2001). Thirdly, injections of 6-OHDA in the GP induced dopamine depletion in both GP and striatum and mimicked the parkinsonian motor symptoms and neural activity abnormalities resulting from striatal 6-OHDA injections (Abedi et al., 2013).

These findings support the important role of dopamine depletion in the GPe for PD. Furthermore, the results of Abedi et al. (2013) additionally indicate that direct injury of the GPe could contribute to PD pathology. Indeed, Fernandez-Suarez et al. (2012) reported prominent cell death of PV-positive GABAergic GPe neurons, commonly projecting to STN and GPi, in 6-OHDA treated rats and in MPTP treated monkeys. In contrast, an earlier study by Hardman and Halliday (1999) did not describe abnormalities in the total number of PV-positive GPe neurons in PD patients. However, when considering cell density rather than absolute cell counts, death of GPe neurons is also possible here as seen in a trend toward a decrease of PV-positive neuron density (Fernandez-Suarez et al., 2012). Fernandez-Suarez et al. (2012) speculate that a loss of GABAergic GPe neurons could decrease inhibition of the STN and thus support its hyperactivity. Furthermore, the GPe may lose parts of its intrinsic structure, thereby forfeiting its ability to perform complex information processing. To prevent secondary cell death, adaptive processes could be triggered that may additionally impede information processing.

## 4. Potential intra-GPe mechanisms for (de)synchronization

Several mechanisms have been proposed that increase synchronization inside the GPe in parkinsonism or, in turn that desynchronize this nucleus under healthy conditions. The majority of these mechanisms are based on interactions between the GPe and other nuclei, namely the STN and striatum (e. g., Alexander and Crutcher, 1990; Ingham et al., 1997; Plenz and Kital, 1999; Terman et al., 2002; Kumar et al., 2011; Fan et al., 2013). In the following sections, we describe intrinsic GPe mechanisms for (de)synchronization, involving cellular and synaptic GPe properties.

### 4.1. Cellular properties

Intrinsic properties of GPe neurons are determined by more than 10 voltage-gated ion channel types (Mercer et al., 2007; Günay et al., 2008; Jaeger and Kita, 2011). Changes in the expression or function of these channels can contribute to changes in activity dynamics and influence synchrony *in vivo*. Hyperpolarization and cyclic nucleotide-gated (HCN), small conductance calcium-activated potassium (SK), and fast, transient, voltage-dependent sodium (NaF) channels as well as general cellular heterogeneity have been proposed for desynchronization of the dopamine intact GPe.

#### 4.1.1. HCN channel expression

HCN channels are widely expressed in the dendrites of neurons in various parts of the brain such as the cortex, hippocampus, and thalamus (Poolos, 2012). They support pacemaking and can take part in sculpting synaptic responses (Chan et al., 2004).

Chan et al. (2004) proposed HCN channels in GP neuron dendrites as key determinants of regular spiking and synchronization. In a study of HCN channel function in 6-OHDA lesioned mice, Chan et al. (2011) uncovered an HCN channelopathy in GP neurons that accompanied pacemaking deficits. HCN channels located presynaptically on GP terminals are known to decrease the likelihood of GABA release (Boyes and Bolam, 2007). Viral delivery of HCN subunits and L-type calcium channel agonists restored pacemaking, but did not improve motor symptoms, suggesting that the channelopathy might therefore be an adaptive process and not causal for motor deficiency.

#### 4.1.2. SK channel expression

SK channels are assumed to contribute to the firing dynamics in most excitable cells (Bond et al., 1999) and can modulate plasticity (Woodward et al., 2010).

Studies with brain slices of healthy rats (Deister et al., 2009) and computational models of GPe neurons (Deister et al., 2009; Schultheiss et al., 2010) proposed a mechanism of decorrelation via an SK current. Deister et al. (2009) showed that rat GP neurons express functional SK channels that contribute to the precision of autonomous firing in GP neurons. Strong SK currents can decrease the sensitivity of GPe neurons to smaller synchronized inputs (Deister et al., 2009). Phase response curve analysis suggests that dendritic SK channel expression controls synchronization by changing the phase dependence of synaptic effects on spike timing (Schultheiss et al., 2010).

SK channels can indirectly be modulated via dopamine (Ramanathan et al., 2008) and may therefore exhibit altered dynamics in PD.

#### 4.1.3. NaF channel expression

The initiation and propagation of action potentials on dendrites significantly depends on NaF channels (Hanson et al., 2004).

High expression of dendritic NaF channels has been suggested as a potential mechanism that actively decorrelates the GPe (Edgerton and Jaeger, 2011). In their computational model, Edgerton and Jaeger (2011) showed that neurons with low dendritic NaF channel expression have a high tendency to phase lock with synchronized synaptic input. They estimated that SK channel expression is only relevant in synchronizing neural activity if the dendritic NaF channel conductance was low compared to the conductance of other channels. Additionally, they report that HCN channel expression did not significantly alter oscillatory firing, leaving dendritic NaF channel expression as the main factor in determining the phase-locking properties of neurons.

GP neurons of rats express dendritic NaF channels and their distribution is enriched near sites of excitatory synaptic input (Hanson et al., 2004). Whether dendritic NaF channel expression actually decreases in PD has not been investigated yet. However, in other neuron types, it has been reported that NaF current density is subject to regulation through multiple pathways and on multiple timescales (Herzog et al., 2003; Hu et al., 2005; Xu et al., 2005), for example by dopamine D_2_ receptor-activated Ca^2+^ signaling within few minutes (Hu et al., 2005).

#### 4.1.4. Cellular heterogeneity

Recently, Deister et al. (2013) suggested cellular heterogeneity as an active decorrelation mechanism. They found that the heterogeneity in firing rates and patterns found in GP neurons in healthy rats are not due to multiple cell types or synaptic transmission but rather caused by a change over time in cellular properties common to all neurons, leading to different cellular characteristics within minutes. Quantitative changes in the expression of HCN or other ion channels could underly this dynamic cellular heterogeneity. Continuous variations in ion channel composition could account for the entire range of firing rates and patterns in the GPe (Günay et al., 2008).

Since neurons firing at widely different rates do not tend to synchronize with each other, this cellular heterogeneity may make the GPe less susceptible to synchronized inputs. Deister et al. (2013) therefore describe a powerful mechanism of decorrelation in the healthy GPe. However, changes in this heterogeneity after dopamine depletion have not yet been investigated.

In summary, in the dopamine intact BG, HCN, SK, and NaF channels as well as cellular heterogeneity have been convincingly argued to contribute to neural dynamics in the GPe. A qualified hypothesis states that GPe neurons are not very dependent on synaptic input due to their intrinsic pacemaker function, potentially sustained by HCN and SK channel function (Wilson, 2013). Loss of the autonomous GPe activity could lead to correlation of neural activity by shared inputs. However, cellular changes in the GPe after dopamine depletion that could cause such a loss are rarely studied in experiments. It therefore seems likely that cellular properties contribute to desynchronization of the healthy GPe, but it remains unclear whether these properties induce synchronization after dopamine depletion.

### 4.2. Synaptic properties

Synaptic coupling inside the GPe via local axon collaterals is well-established (Francois et al., 1984; Kita, 1994; Sadek et al., 2007; Miguelez et al., 2012) although functional GPe connectivity is highly variable and depends on the brain state (Magill et al., 2006). Rat GP-GP synapses have considerably different properties than striatum-GP synapses, with a lower paired-pulse ratio and weak responses to stimulation (Sims et al., 2008).

Although little is known about the effects of GABAergic transmission within the GPe, connections from the GPe to the STN and the substantia nigra pars reticulata (SNr) are better described and may share characteristics of GPe-GPe connections. In rat brain slices, GP-STN connections have been found to be sparse, but sufficiently powerful to inhibit and synchronize the autonomous activity of STN neurons (Baufreton et al., 2009). Bursts of activity from the rat GP are also able to effectively silence the firing of SNr neurons, although they can start firing again due to depression of these GP-SNr synapses (Connelly et al., 2010). A recent study demonstrates that GP-GP connections are also highly efficacious in modulating postsynaptic activity despite substantial short time depression and sparse connectivity (Bugaysen et al., 2013).

#### 4.2.1. Synaptic strength

Miguelez et al. (2012) showed that GP-GP inhibitory synaptic transmission increased in a rat 6-OHDA model, leading to increased rebound bursting. This altered transition may have major impacts on neural dynamics. Kita et al. (2004, 2006) demonstrated that specific blocking of GABA receptors in the monkey GPe regularizes neuron firing, indicating that GABAergic inhibition from the striatum and GPe regulates pallidal firing.

It is still unclear how much and which influence inhibitory GPe-GPe coupling has on synchrony in parkinsonism. If their firing is periodic, coupling between GPe cells could either synchronize or desynchronize activity (Wilson, 2013). In the healthy GPe, given the pacemaking function of these neurons, local axon collaterals may act as desynchronizing elements (Sims et al., 2008; Wilson, 2013). However, after dopamine depletion, the effect of local axon collaterals could be reversed and synchronize activity (Wilson, 2013).

#### 4.2.2. Synaptic architecture

Highly heterogeneous synaptic coupling between GPe neurons could be a factor for their desynchronization. As heterogeneity on a cellular basis can act as a decorrelator, highly inhomogeneous coupling amongst neurons could lead to similar effects. Sadek et al. (2007) described the anatomical network of GP-GP axon collaterals in the rat as structured rather than homogeneously distributed. It can be speculated that through injury or adaptive remodeling, this structure may become damaged and lose the ability to desynchronize.

Although changes in synaptic transmission within the rat GP after dopamine depletion have been measured (Miguelez et al., 2012), the detailed intrinsic connectivity of GPe still remains poorly understood. Nevertheless, it has become evident that this nucleus cannot only be considered as a homogeneous relay nucleus (Sadek et al., 2007). Further studies of its structural and functional connectivity, especially at different dopamine levels, are needed to shed light on information processing inside the GPe.

## 5. Conclusions

Several lines of evidence emphasize the importance of intrinsic GPe properties in abnormal synchronization in parkinsonism. This makes the GPe an attractive target for future therapies, potentially involving direct pharmacological targeting.

Most of the evidence provided in this review is based on rodent studies, but the rodent GP may differ substantially from the human GPe in some aspects. Functionally, a lower average firing rate has been observed in the rodent GP compared to the primate GPe, while firing patterns were very similar (Benhamou et al., 2012). Anatomically, little is known about the level of human GPe local collateralization, although its existence is hardly debated (Francois et al., 1984). The rat GP is studied in more detail and shows a high level of complex local connections (Sadek et al., 2005, 2007).

Though often assumed, it remains unclear whether increased synchronization in the BG causes motor impairments in PD patients (Quiroga-Varela et al., 2013). The onset of the synchronization process occurs independently of the onset of motor symptoms in animal models of increasing levels of dopamine depletion (Leblois et al., 2007; Dejean et al., 2012). Nevertheless, impact of β band synchronization on motor control remains an established assumption (Brittain and Brown, 2013).

A comprehensive mechanism responsible for synchronization and desynchronization of the GPe that is dependent on dopamine levels, is still missing. However, loss of pacemaker function in GPe neurons and altered function of GPe-GPe synapses are important candidates (Wilson, 2013). Although this review focuses on intrinsic GPe properties, we do not suggest that interactions in the BG network are less important. Synaptic input to the GPe, mainly from the STN, plays a major role in pallidal synchronization (Goldberg et al., 2003; Tachibana et al., 2011). We propose intrinsic mechanisms of the GPe as crucial in processing these synchronized or partly synchronized inputs.

After dopamine depletion, GP neurons undergo plastic changes in their synaptic and cellular structure (Chan et al., 2011; Miguelez et al., 2012; Wichmann and Smith, 2013), which may potentially trigger synchronized neural activity. However, further studies on ion channel remodeling after dopamine depletion and their effects on synchrony and motor performance are missing. Intrinsic GPe connectivity is still insufficiently described and may not be restricted to GABAergic transmission. We emphasize that special attention should be drawn to possible cell death in the GPe (Fernandez-Suarez et al., 2012). Adaptive processes could be triggered to prevent further cell death that may lead to altered neural activity, which might involve synaptic as well as cellular changes.

### Conflict of interest statement

The authors declare that the research was conducted in the absence of any commercial or financial relationships that could be construed as a potential conflict of interest.

## Footnotes

^1^The primate external and internal globus pallidus (GPe and GPi) are named globus pallidus (GP) and endopenduncular nucleus (EP), respectively, in rodents. We will refer to GPe and GPi for these nuclei in general.
